# Risk factors associated with venous thromboembolism in tuberculosis: A case control study

**DOI:** 10.1111/crj.13555

**Published:** 2022-11-07

**Authors:** Guo Yi Nan, He Fei, Wang Zhen, Duan Tian Yun

**Affiliations:** ^1^ Department of Tuberculosis, Affiliated Hangzhou Chest Hospital Zhejiang University School of Medicine Hangzhou China; ^2^ Department of Respiratory diseases, Affiliated Hangzhou Chest Hospital Zhejiang University School of Medicinel Hangzhou China; ^3^ Department of medical quality control, Affiliated Hangzhou Chest Hospital Zhejiang University School of Medicine Hangzhou China; ^4^ Department of Thoracic Surgery, Sir Run Run Shaw Hospital Zhejiang University School of Medicine Hangzhou China

**Keywords:** correlation, risk factors, tuberculosis, venous thromboembolism

## Abstract

Tuberculosis (TB) patients who develop venous thromboembolism (VTE) have poor clinical outcomes. This study aimed to explore the risk factors and the prognosis of TB patients with VTE. A total of 11 267 with TB patients from the Zhejiang University‐affiliated Chest Hospital, China, were enrolled between January 2016 and January 2020. A total of 107 TB patients with VTE were selected as the VTE group. Patients in the control group were randomly screened in a 1:1 ratio between the VTE and control groups. Univariate and multivariate logistic regression analysis was used to evaluate the factors associated with VTE in TB patients. Of the 214 patients, 145 (60.17%) were male, 69 (32.2%) were female, with an average age of 62.21 ± 19.35. The incidence of VTE in TB patients was 0.95%. Using a univariate analysis, it was found that age, fever, dyspnea, lower limb edema, respiratory failure, malignant tumor, prothrombin time, activated partial thromboplastin time, D—dimer, and hemoglobin levels were different between the two groups (*P* < 0.05). Multivariate logistic regression analysis showed that higher D‐dimer value, higher incidence of lower limb edema, and TB were risk factors for VTE; OR (95%CI) = 8.840 (2.383–32.794); OR (95%CI) = 4.957 (1.219–20.161); OR (95%CI) = 16.216 (4.779–55.025). However, the use of Rifamycin was found to be a protective factor against VTE [OR (95%CI) = 0.170(0.073–0.395)]. Receiver operating characteristic curve (ROC) curve of D‐dimer (area under curve [AUC] = 0.831 ± 0.028 [95%CI: 0.776–0.886, *P* < 0.05]) and the cut‐off value of 1855 μg/L was obtained according to the Youden index, with a sensitivity and a specificity rate of 82.2% and 74.3%, respectively. The risks of VTE seem higher in TB patients with fever, dyspnea, lower limb edema, and D‐dimer levels of more than 1855 μg/L; therefore, it should be actively screened, and prophylactic anticoagulation given if necessary. Effective directly observed treatment plus short‐course chemotherapy (DOTS) protocol anti‐TB therapy helps reduce the probability of VTE in TB patients.

## Graphical abstract

Venous thromboembolism (VTE) risks seem higher in tuberculosis (TB) patients, and therefore, there is a need for active prevention and treatment. Patients with fever, dyspnea, lower limb edema, and D‐dimer levels of more than 1855 μg/L should be actively screened for VTE and prophylactic anticoagulation given if necessary. Effective directly observed treatment plus short‐course chemotherapy (DOTS) protocol anti‐TB therapy helps reduce the probability of VTE in TB patients.

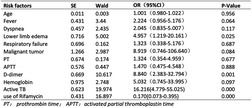

## 1 INTRODUCTION

Venous thromboembolism (VTE) is the third most common cardiovascular disease associated with higher morbidity and mortality.^1^ Globally, tuberculosis (TB) is a serious infectious disease that mainly affects the lung and, if not treated well, is carried with high mortality, especially in developing countries. It is also one of the preventable infectious diseases that are common in China.^2^

Many studies have shown that TB patients are more likely to suffer from VTE, and the mortality is as high as 15%.^3^, ^4^ So the key to reducing VTE incidence in TB patients is identifying high‐risk patients and giving them timely prophylactic treatments. Consequently, this study aims to analyze the factors associated with the development of VTE in TB patients and its prognosis.

## 2 METHODOLOGY

### 2.1 Study population and design

This study was conducted at the Zhejiang University‐affiliated Chest Hospital, China. We retrospectively analyzed all inpatient cases diagnosed with a clinically confirmed TB^5^ between January 2016 and January 2020. We excluded TB patients who stayed in the hospital for only 1 day and those with surgical treatments.

A total of 107 TB patients with VTE were identified.^6^ On the other hand, samples for the controls consisted of TB patients without VTE, in which one control was assigned for each case (107 cases). Controls were selected by random number table, totaling 214 patients (see supporting information Figure S1). We selected risk factors for TB patients with VTE based on previous studies from an accessible and reliable clinical database. Records were extracted from the patients' electronic medical records database, including age, sex, clinical symptoms, physical exam findings, smoking and alcohol intake, comorbidities, clinical laboratory findings, the severity of TB, and treatment history (Rifamycin).

### 2.2 Definitions

Active TB was defined as follows: (1) pulmonary TB: *Mycobacterium tuberculosis* complex was identified by Biochip assays using sputum specimens with acid‐fast bacilli that were smear‐positive or culture‐positive. (2) Extrapulmonary TB (EPTB): patients with at least one specimen extracted from infected lesions other than the lung that was a confirmed *M. tuberculosis* infection using biochip assays. Rifamycin therapy was defined as the inpatient use of at least 3 days before or after hospitalization.

### 2.3 Statistical analysis

The data were entered into SPSS for Windows, version 24(SPSS I nc, USA). Continuous data were described as ^−^X ± s. Continuous variables were analyzed with the independent samples *t*‐test. Categorical variables were compared with Pearson's Chi‐square test. We included variables with a *p‐*value of <0.05 into a multivariate logistic regression model with stepwise forward selection to identify independent risk factors. Then, we used the receiver operating characteristic curve (ROC) to describe the continuous variables with statistical significance and derive the cut‐off value per the Youden index. The criterion for statistical significance was *p* < 0.05.

### 2.4 Ethics approval

This study was approved by the Hangzhou Red Cross Hospital Ethics Committee (2020) Quick review no. 187).

## 3 RESULTS

A total of 11 267 patients were included in the study, with 7713 males (68.5%) and 3554 females (31.5%). Of this, 107 patients had confirmed concurrent TB with VTE. The incidence of VTE in TB patients was 0.95%. The median age of all patients included in the study was 62.21 ± 19.35 years; however, those with VTE were older (median age 65.78 ± 16.66；*p* = 0.007) as compared with those without VTE (median age 58.64 ± 21.19).

**FIGURE 1:**
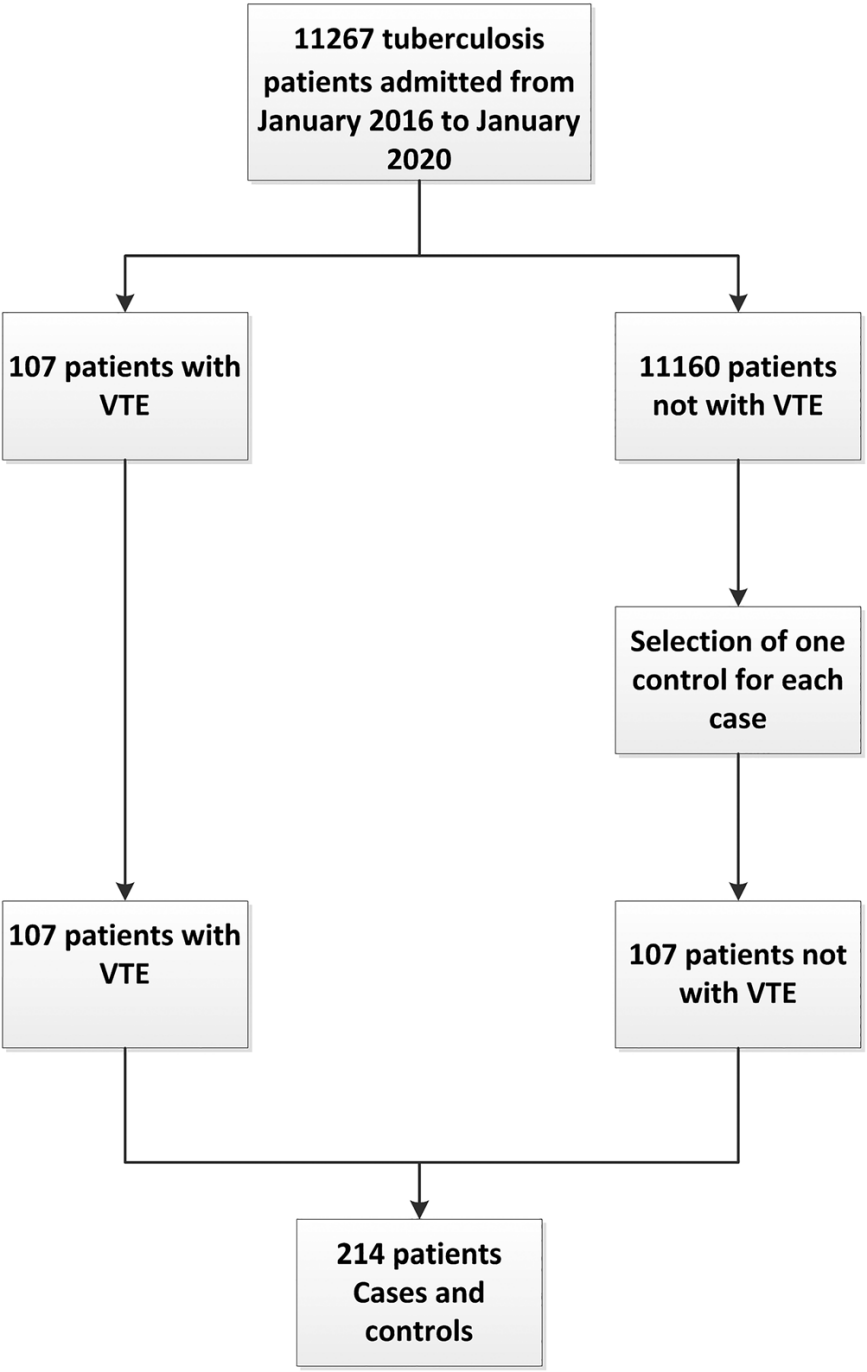
Flowchart of study recruitment and screening process of the cases and controls

In total, 68 (31.78%) patients had a fever, of which 45 (66.18%) were confirmed with VTE. Analysis showed that patients with VTE were more likely to have fever (*p* = 0.001). Those with dyspnea were 67 (31.31), of which 41 (61.19%) were confirmed with VTE. Similarly, the analysis showed that patients with dyspnea were more likely to have VTE (*p* = 0.027). A total of 27 (12.62%) patients had lower limb edema, and 22 (81.48%) confirmed with VTE. Likewise, patients with lower limb edema were more likely to be diagnosed with VTE (*p =* 0.000). Lastly, respiratory failure (*p* = 0.007) and malignant tumor (*p* = 0.017) had a statistically significant association with VTE occurrence in TB patients (Figure 1).

Univariate analysis suggested that no significant differences were observed between the VTE group and the control group in the distribution of age, gender, cough, smoking and alcohol status, hemoptysis, varicose veins of the lower limbs, and comorbidities such as diabetes, coronary heart disease, chronic obstructive pulmonary disease, nephrotic syndrome, heart failure, and ischemia stroke. The details of comparing the clinical characteristics of patients with and without VTE are summarized in Table 1.

**TABLE 1:** General information in two groups

Characteristics	VTE group (*n* = 107)	Control group (*n* = 107)	Statistic	*P*‐value
**Age (X ± s)**	65.78 ± 16.66	58.64 ± 21.19	−2.736	0.007
**Gender (*n*, %)**				
Male	78 (72.9%)	67 (62.6%)	2.588	0.108
Female	29 (27.1%)	40 (37.4%)		
Smoking	52 (48.6%)	41 (38.3%)	2.301	0.129
Alcohol	32 (29.9%)	27 (25.2%)	0.585	0.444
**Clinical symptoms**				
Cough	70 (65.4%)	57 (53.3%)	3.273	0.07
Hemoptysis	6 (5.6%)	11 (10.3%)	1.597	0.206
Fever	45 (42.1%)	23 (21.5%)	10.433	0.001
Chest pain	6 (5.6%)	13 (12.1%)	2.83	0.093
Dyspnea	41 (38.3%)	26 (24.3%)	4.889	0.027
**Physical exam findings**				
Lower limb edema	22 (20.6%)	5 (4.7%)	12.249	0.000
**Comorbidities**				
Varicose veins of lower limb	2 (1.9%)	0 (0%)	0.505	0.477^a^
Diabetes	15 (14.0%)	17 (15.9%)	0.147	0.701
Hypertension	34 (31.8%)	36 (33.6%)	0.085	0.771
Coronary heart disease	13 (12.1%)	10 (9.3%)	0.438	0.508
COPD	13 (12.1%)	14 (13.1%)	0.042	0.837
Nephrotic syndrome	2 (1.9%)	2 (1.9%)	0	1.000
Respiratory failure	17 (15.9%)	5 (4.9%)	7.298	0.007
Heart failure	6 (5.6%)	1 (0.9%)	3.692	0.055
Ischemia stroke	5 (4.7%)	0 (0%)	3.277	0.07
Myocardial infarction	0 (0%)	0 (0%)		1.0
Acute infectious diseases	1 (0.9%)	0 (0%)	0	1.0
Malignant tumor	10 (9.3%)	2 (1.9%)	5.65	0.017

Abbreviations: COPD, chronic obstructive pulmonary disease; VTE, venous thromboembolism.

^a^ Method of successive corrections.

Due to some unknown reason, the results of the routine blood test of one patient and the coagulation profile of another patient were both missing. The laboratory findings showed that the PT (*p* = 0.001), APTT (*p* = 0.000), and D‐dimer (*p* = 0.000) were all significantly higher in the VTE group than in the control group. However, the hemoglobin level (Hb, *p* = 0.000) was significantly lower in the VTE group than in the control group. White blood cells and platelet count were not associated with the occurrence of VTE (Tables 2 and 3).

**TABLE 2:** Coagulation profile of the two groups

Laboratory findings	VTE group (*n =* 107)	Control group (*n =* 106)	Statistic	*P*‐value
**PT** (**s)**				
>14.3	22 (20.6%)	6 (5.7%)	10.355	0.001
≤14.3	85 (79.4%)	100 (94.3%)		
**APTT** (**s)**				
>34	33 (30.8%)	12 (11.3%)	12.177	0.000
≤34	74 (69.2%)	94 (88.7%)		
**D‐dimer** (**μg/L)**				
>550	104 (97.2%)	64 (60.4%)	42.327	0.000
≤550	3 (2.8%)	41 (39.6%)		

Abbreviations: APTT, activated partial thromboplastin time; PT, prothrombin time; VTE, venous thromboembolism.

**TABLE 3:** Routine blood test results of the two groups

Laboratory findings	VTE group (*n =* 106)	Control group (*n =* 107)	Statistic	*P*‐value
**WBC** (*10^9)				
>9.5	21 (19.8%)	15 (14.1%)	1.457	0.483
3.5–9.5	77 (72.7%)	85 (79.4%)		
<3.5	8 (7.5%)	7 (6.5%)		
**Hemoglobin** (**g/L)**				
>170	2 (1.9%)	10 (9.3%)	23.442	0.000
120–170	42 (39.6%)	68 (63.6%)		
<120	62 (58.5%)	29 (27.1%)		
**Platelet**				
>300	24 (22.6%)	12 (11.2%)	5.267	0.072
100–300	76 (71.7%)	90 (84.1%)		
<100	6 (5.7%)	5 (4.7%)		

Abbreviations: VTE, venous thromboembolism. WBC, white blood cells.

Active TB as a disease was associated with the occurrence of VTE (*p* = 0.001), while the use of Rifamycin reduced the risks of VTE occurrence (*p* = 0.000). The anatomical sites of TB were not associated with VTE risks (Table 4).

**TABLE 4:** Active tuberculosis as use of Rifamycin between the two groups

Characteristics	VTE group (*n =* 107)	Control group (*n =* 107)	Statistic	*P*‐value
Active TB	99 (92.5%)	81 (75.7%)	11.329	0.001
**Anatomical site of TB**				
PTB	75 (70.1%)	77 (72.0%)	0.226	0.893
EPTB	21 (19.6%)	21 (19.6%)		
Both	11 (10.3%)	9 (8.4%)		
Use of Rifamycin	39 (36.4%)	70 (65.4%)	17.969	0.000

Abbreviations: EPTB, extrapulmonary tuberculosis; PTB, pulmonary tuberculosis; TB, tuberculosis; VTE, venous thromboembolism.

Multivariate logistic regression analysis revealed that high level of D‐dimer (OR: 8.84; 95%CI: 2.383–32.794, *p* = 0.001), lower limb edema (OR: 4.957; 95%CI: 1.219–20.161, *p* = 0.025), and active TB (OR: 16.216; 95%CI: 4.779–55.025, *p* = 0.000) all contributed to occurrence of VTE in TB patients (Table 5).

**TABLE 5:** Risk factors of VTE in TB patients

Risk factors	SE	Wald	OR (95%CI)	*P*‐value
Age	0.011	0.003	1.001 (0.980–1.022)	0.956
Fever	0.431	3.44	2.224 (0.956–5.176)	0.064
Dyspnea	0.457	2.435	2.045 (0.835–5.007)	0.117
Lower limb edema	0.716	5.002	4.957 (1.219–20.161)	0.025
Respiratory failure	0.696	0.162	1.323 (0.338–5.176)	0.687
Malignant tumor	1.266	2.987	8.919 (0.746–106.640)	0.084
PT	0.674	0.174	1.324 (0.354–4.959)	0.677
APTT	0.576	0.447	1.470 (0.475–4.548)	0.888
D‐dimer	0.669	10.617	8.840 (2.383–32.794)	0.001
Hemoglobin	0.975	2.748	5.032 (0.745–33.995)	0.097
Active TB	0.623	19.974	16.216 (4.779–55.025)	0.000
use of Rifamycin	0.431	16.897	0.170 (0.073–0.395)	0.000

Abbreviaitons: APTT, activated partial thromboplastin time; PT, prothrombin time； TB, tuberculosis; VTE, venous thromboembolism.

**FIGURE 2:**
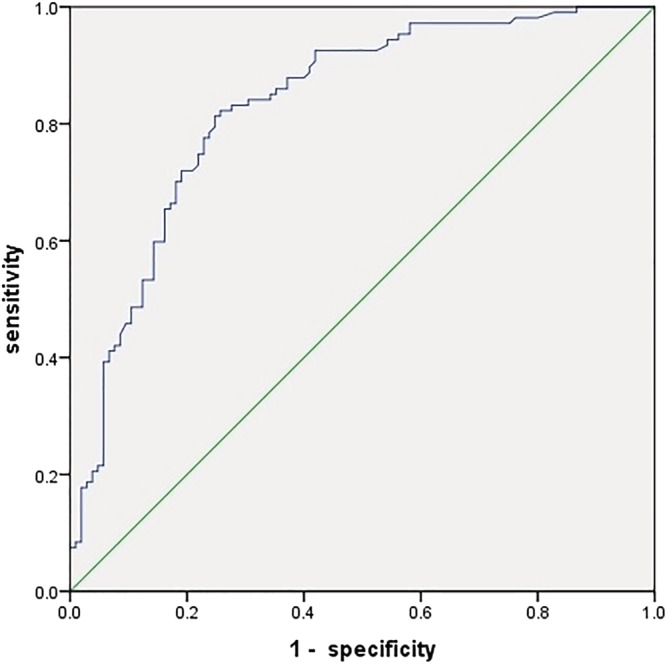
Receiver operating characteristic curve (ROC) curve of D‐dimer

D‐dimer was analyzed using the ROC (Figure 2). The results showed that D‐dimer levels differed significantly between the two groups (area under curve [AUC] = 0.831 ± 0.028 [95%CI: 0.776–0.886], *p* < 0.05). The cut‐off value was 1855 μg/L, as calculated by the Youden index, with a sensitivity of 82.2% and a specificity of 74.3%.

## 4 DISCUSSION

Epidemiological studies estimate the incidence of VTE to be around 0.1% to 0.2% in the general population and 0.7% to 3.9% in patients with active pulmonary TB.^7^, ^8^, ^9^ In this study, the incidence of VTE was 0.95%, which is consistent with previously reported studies. These data show that TB patients have a higher risk of VTE than the general population, and physicians should be educated on the prevention and treatment of VTE in TB patients.

In our analysis, patients in the VTE group were older than those in the control group, which indicates that older age could be at a higher risk of developing VTE. However, older patients generally have other comorbidities, and sometimes their symptoms could overlap with VTE. Other studies also show that the clinical manifestations of VTE are usually diverse and sometimes nonspecific; hence, misdiagnosis and treatment delay are common.^10^ We also found that VTE easily complicated clinical symptoms such as fever, dyspnea, and lower limb edema.

Hypercoagulability is one component of the Virchow triad. There is evidence that TB patients are in a hypercoagulable state with a higher D‐dimer level than the general population.^11^ In clinical practice, if the plasma D‐dimer is negative, VTE can be largely excluded; however, D‐dimer has a poor positive predictive value, mainly because it can also be higher in patients with active inflammation, malignancy, or infectious processes.^12^ Nonetheless, some studies show that elevated D‐dimer levels, despite being nonspecific, still guide clinical evaluation in patients with suspected VTE.^13^ D‐dimer was found to be a risk factor for VTE in TB patients (OR 8.84, 95%CI: 2.383–32.794, *p* < 0.05), with an AUC value of 0.78–0.89 and a cut‐off value of 1855 μg/L. Therefore, clinicians should pay more attention to patients with a high D‐dimer level, especially if higher than 1855 μg/L.

Currently, the mechanisms underlying the increased risk of VTE in TB patients remain unknown. Some researches^14^, ^15^ proposed that the chronicity of TB infection might cause vascular endothelial cell remodeling and subsequent release of numerous inflammatory mediators and cytokines such as IL‐6 and TNFα. These cytokines further cause the production of various proteins of acute inflammation and coagulation factors, while simultaneously inhibiting fibrinolysis by downgrading antithrombin III, free protein C, protein S, and protein C.

White et al.^16^ found that, compared with other anti‐TB regimens, the risk of DVT was higher in Rifamycin containing regimen (OR 4.74). Conversely, we found rifamycin use as a protective factor in which it reduced the occurrence of VTE. The reason may be that Rifamycin has a strong anti‐TB effect.

Moreover, the data by White and colleagues are from the period between 1978 and 1986, an era in which short‐course therapy with isoniazid and rifampicin was the standard of care, as compared with modern‐day directly observed treatment plus short‐course chemotherapy (DOTS); therefore, absolute causation of Rifamycin with VTE cannot be concluded. Similarly, our study is a retrospective study with a small sample size, and therefore, larger studies are needed to confirm the effect of Rifamycin in TB patients with VTE.

## 5 CONCLUSION

VTE risks seem higher in TB patients, and therefore, there is a need for active prevention and treatment. Patients with fever, dyspnea, lower limb edema, and D‐dimer levels of more than 1855 μg/L should be actively screened for VTE and prophylactic anticoagulation given if necessary. Effective DOTS protocol anti‐TB therapy helps reduce the probability of VTE in TB patients.

## CONFLICT OF INTEREST

The authors declare no conflict of interest. The manuscript has been read and approved by all the authors and that each author believes that the manuscript represents honest work.

## ETHICS STATEMENT

The permission was obtained for the study from the Local Ethics Committee of Affiliated Hangzhou Chest Hospital, Zhejiang University School of Medicine. This study was exempted from signing patient consent statement, because this study only uses patient data with identifiable information and the research project does not involve personal privacy and commercial interests.

## AUTHOR CONTRIBUTIONS

Guo Yi Nan collected patients' data and carried out statistical analysis and article writing. He Fei wrote the original manuscript; Wang Zhen offered guidelines for the research design; Duan Tian Yun: research design idea, proofreading, and offered guidance on statistical analysis.

## Supporting information




**Data S1.** Supporting Information

## Data Availability

The data set used and/or analyzed during the current study are available from the corresponding author on reasonable request.
