# Supplementary figures and images for: Risk factors associated with venous thromboembolism in tuberculosis: A case control study
Source: Clin Respir J. 2022 Nov 7;16(12):835–41. doi: 10.1111/crj.13555 (PMC9716713; doi:10.1111/crj.13555)

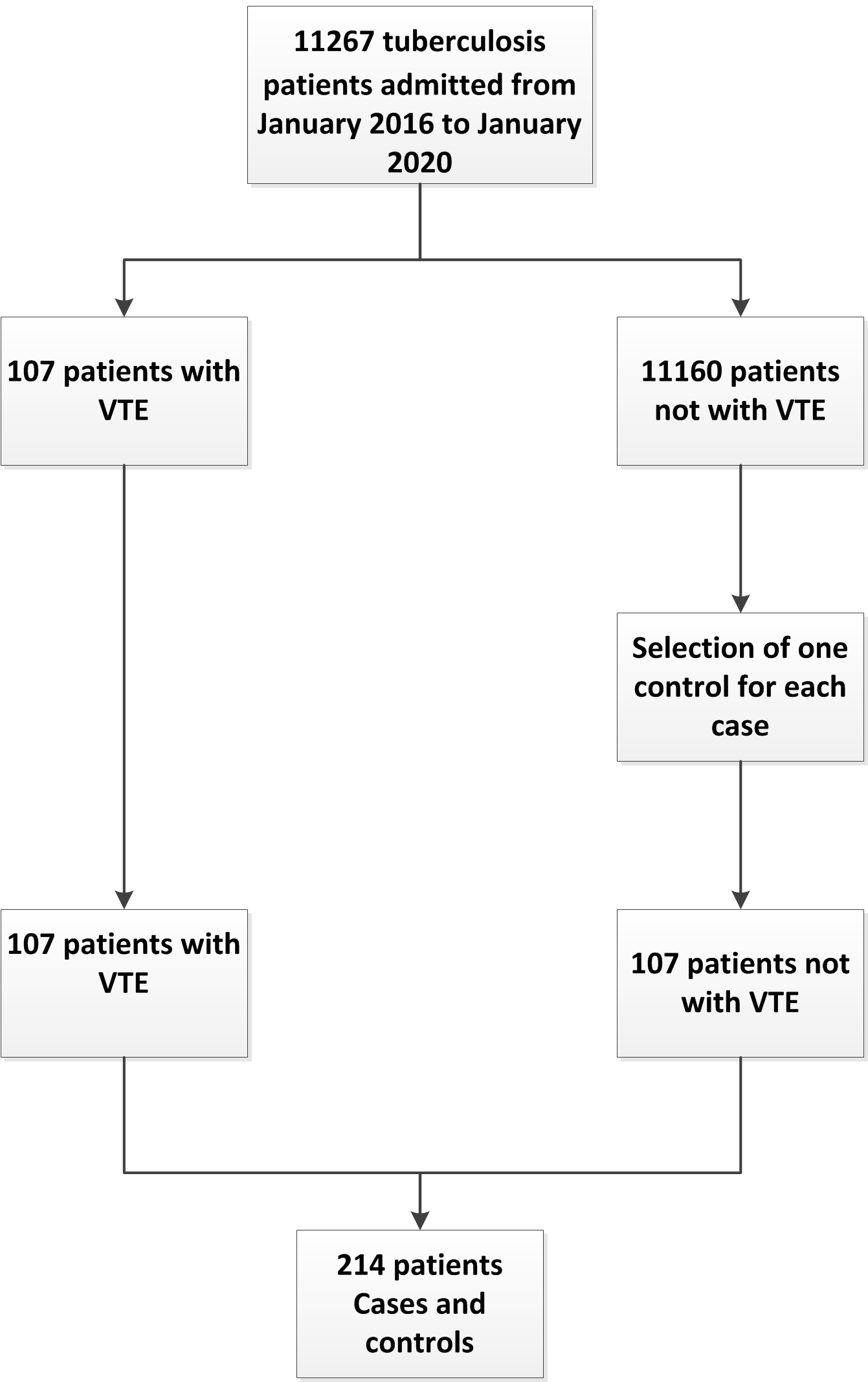

Supplement: Supplementary file 1 — Data S1. Supporting Information [file CRJ-16-835-s001.tif]
